# Pathological phosphorylation of tau and TDP-43 by TTBK1 and TTBK2 drives neurodegeneration

**DOI:** 10.1186/s13024-018-0237-9

**Published:** 2018-02-06

**Authors:** Laura M. Taylor, Pamela J. McMillan, Nicole F. Liachko, Timothy J. Strovas, Bernardino Ghetti, Thomas D. Bird, C. Dirk Keene, Brian C. Kraemer

**Affiliations:** 1 Geriatrics Research Education and Clinical Center, Veterans Affairs Puget Sound Health Care System, S182, 1660 South Columbian Way, Seattle, WA 98108 USA; 2 Department of Psychiatry and Behavioral Sciences, University of Washington, Seattle, Washington, 98195 USA; 3 Division of Gerontology and Geriatric Medicine, Department of Medicine, University of Washington, Seattle, WA 98104 USA; 4 Department of Pathology and Laboratory Medicine, Indiana University School of Medicine, Indianapolis, IN 46202 USA; 5 Department of Neurology, University of Washington, Seattle, WA 98195 USA; 6 Division of Medical Genetics, Department of Medicine, University of Washington, Seattle, WA 98104 USA; 7 Department of Pathology, University of Washington, Seattle, WA 98195 USA

**Keywords:** Frontotemporal lobar degeneration, TTBK1, TTBK2, TDP-43, Tau, *C. elegans*, Neurodegeneration

## Abstract

**Background:**

Progressive neuron loss in the frontal and temporal lobes of the cerebral cortex typifies frontotemporal lobar degeneration (FTLD). FTLD sub types are classified on the basis of neuronal aggregated protein deposits, typically containing either aberrantly phosphorylated TDP-43 or tau. Our recent work demonstrated that tau tubulin kinases 1 and 2 (TTBK1/2) robustly phosphorylate TDP-43 and co-localize with phosphorylated TDP-43 in human postmortem neurons from FTLD patients. Both TTBK1 and TTBK2 were initially identified as tau kinases and TTBK1 has been shown to phosphorylate tau epitopes commonly observed in Alzheimer’s disease and other tauopathies.

**Methods:**

To further elucidate how TTBK1/2 activity contributes to both TDP-43 and tau phosphorylation in the context of the neurodegeneration seen in FTLD, we examined the consequences of elevated human TTBK1/2 kinase expression in transgenic animal models of disease.

**Results:**

We show that *C. elegans* co-expressing tau/TTBK1 tau/TTBK2, or TDP-43/TTBK1 transgenes in combination exhibit synergistic exacerbation of behavioral abnormalities and increased pathological protein phosphorylation. We also show that *C. elegans* co-expressing tau/TTBK1 or tau/TTBK2 transgenes in combination exhibit aberrant neuronal architecture and neuron loss. Surprisingly, the TTBK2/TDP-43 transgenic combination showed no exacerbation of TDP-43 proteinopathy related phenotypes. Additionally, we observed elevated TTBK1/2 protein expression in cortical and hippocampal neurons of FTLD-tau and FTLD-TDP cases relative to normal controls.

**Conclusions:**

Our findings suggest a possible etiology for the two most common FTLD subtypes through a kinase activation driven mechanism of neurodegeneration.

**Electronic supplementary material:**

The online version of this article (10.1186/s13024-018-0237-9) contains supplementary material, which is available to authorized users.

## Background

Frontotemporal lobar degeneration (FTLD) is a progressive neurodegenerative disease clinically diagnosed by evidence of personality and behavioral changes and language dysfunction [1]. Following Alzheimer’s disease (AD), FTLD is the second most prevalent form of presenile dementia affecting 10-30 per 100,000 individuals between the ages of 45 and 65 years. In general, FTLD features atrophy of the frontal and temporal lobes resulting from neuron loss [2]. FTLD is sub-classified into three major pathological subtypes based on the presence of aggregated protein deposits of either TDP-43 or tau inclusions, with a small subset of cases exhibiting FUS-related pathology [2]. FTLD-TDP accounts for roughly 50% of cases whereas FTLD-tau accounts for approximately 45% of cases [2]. FTLD-TDP presents with aberrantly processed, ubiquitinated, and phosphorylated TDP-43 in neuronal inclusions and dystrophic neurites. FTLD-tau, on the other hand, is characterized by hyperphosphorylated aggregates of tau, which form tangles and pick bodies in neurons, glia, and neurites. Several mutations in *MAPT* reduce tau’s affinity for microtubules and increase its aggregation rate and can cause FTLD-tau [3].

Phosphorylation of TDP-43 at serine residues 409 and 410 remains a consistent pathological feature in ALS and FTLD-TDP [4]. Phosphorylation of TDP-43 reduces TDP-43 protein turnover, increases cellular mislocalization of TDP-43, drives protein aggregation, and promotes neurodegeneration [5–9]. Likewise, hyperphosphorylated tau protein is a hallmark of several neurodegenerative disorders including AD, progressive supranuclear palsy (PSP), corticobasal degeneration (CBD), and FTLD. Phosphorylated tau has been implicated in the formation of toxic tau aggregates that promote neurodegeneration [10–15].

The kinases TTBK1 and TTBK2 have been implicated in a number of neurodegenerative diseases. TTBK1 protein is increased in AD and influences the aggregation of tau [16, 17]. Furthermore, transgenic mouse lines expressing full-length human TTBK1 exhibit age-dependent detriments consistent with neurodegeneration, including learning impairment, neurofilament aggregation, microgliosis, altered CDK5/p35 activity, and decreased expression of NMDA receptors [18]. A double-transgenic mouse model expressing FTLD mutant tau and human TTBK1 shows increased accumulation of oligomeric tau and enhanced motor neuron loss, suggesting a direct role of TTBK1 in accelerating tau-related neurodegeneration [19]. Mutations in TTBK2 cause spinocerebellar ataxia type 11, a disorder exhibiting both loss of Purkinje cells and widespread deposition of tau [20]. TTBK2 plays an essential role in the initiation of ciliogenesis during embryonic development through the regulation of microtubule dynamics [21–23]. TTBK1 is solely expressed in the CNS and reproductive tissues, whereas TTBK2 is ubiquitously expressed throughout many tissues including liver, skeletal muscle, pancreas, heart, and brain [24].

Purified recombinant human TTBK1 and TTBK2 can directly phosphorylate both TDP-43 at S409/410 [9] and tau at S198, S199, S202, and S422 [17] and S208 and 210 [25]. Additionally, both TTBK1 and TTBK2 co-localize with phosphorylated TDP-43 (pTDP) in human postmortem tissues from both FTLD and ALS cases [9] and phosphorylated tau (ptau) in AD cases [16]. Since TTBK1/2 phosphorylate both TDP-43 and tau, a common pathway could be involved in the initiation of FTLD. While TTBK1/2 directly phosphorylate TDP-43 and tau in vitro*,* it remains unclear how TTBK1/2 activity in vivo influences disease onset and progression.

To examine how TTBK1/2 contribute to both TDP-43 and tau phosphorylation, we analyzed their effects on lifespan, proteostatic function, and neurodegeneration in the context of tau or TDP-43 transgenic animal models. We also examined the expression of TTBK1 and TTBK2 in post-mortem human brain. We demonstrate that TTBK1/2 kinase expression leads to significant neurodegenerative phenotypes in our transgenic tau and TDP-43 models and is also a consistent feature of FTLD-tau and FTLD-TDP.

## Methods

### *C. elegans* Strains

The N2 (Bristol) strain of *C. elegans* was used for all experimental controls and maintained as described [26]. Strains were maintained at 20 °C on OP50 seeded nematode growth media (NGM). All experiments were performed at room temperature unless otherwise designated. Construction and characterization of TDP-43 transgenic (tg) (CK410) and tau (high-expression) (CK144) lines used were described previously [8]. Tau (low-expression) (CK1044) tg strains were generated by introducing wild type human full length 1N4R splice isoform tau cDNAs driven by the pan neuronal *aex-3* promoter (*Paex-3*::Tau) into the *C. elegans* genome.

TTBK1 and TTBK2 kinase domain (hTTBK1-cat and hTTBK2-cat) strains were constructed by introducing human TTBK1 or TTBK2 cDNA encoding the kinase domain, driven by the pan neuronal *rgef-1* promoter (*Prgef-1*::hTTBK1cat, *Prgef-1*::hTTBK2cat) (TTBK1:CK1051; TTBK2:CK646, CK645) into the *C. elegans* genome. *Prgef-1*::hTTBK1cat was microinjected into N2 at a concentration of 30 ng/μl with an *elt-2*::mCherry coinjection marker at a concentration of 25 ng/μl. *Prgef-1*::hTTBK2cat was microinjected into N2 at a concentration of 50 ng/μl with an *elt-2*::mCherry coinjection marker at a concentration of 25 ng/μl. For all transgenic strains, extrachromosomal arrays were then integrated by exposing animals to a dose of 4000 Rad Gamma rays and subsequently outcrossed back into the N2 background at least twice. TTBK1 and TTBK2 strains used were CK1051, CK645, CK646. Strain CZ1200 [27], which carries an integrated *Punc25::GFP* transgene marker in GABAergic motor neurons, was a generous gift from Dr. Y. Jin. CK1051 and CK646 were crossed with CK1044, CK144, and CK410 to generate homozygous double transgenic *C. elegans*. CK646/CK1044 and CK1051/CK144 double transgenic were subsequently crossed with CZ1200 to produce triple transgenic lines with GFP marked GABAergic neurons.

### Radial locomotion assay

Behavior was assessed by placing 15-20 age-matched (L4) *C. elegans* at the center of a 150 mm NGM plate supplemented with 5× peptone (5xPEP), with a uniform OP50 bacterial lawn. After 1 h of free movement at room temperature, the radial distance traveled from the origin by each animal was measured. Distance from the origin traveled per unit of time was expressed in micrometers per second to give a radial velocity. The assay was performed in triplicate by an observer blinded to genotype and statistical analyses were performed using GraphPad Prism software.

### Lethality assay

CK144 was crossed with CK646 or CK645 and made homozygous for either the tau or hTTBK2-cat transgene to create the F1 population. From a single F1 parent, each F2 progeny was isolated onto an individual plate and the subsequent F3 offspring were scored for transgene expression. Animals that did not survive past L2 were characterized as larval lethal. Animals that survived into adulthood but did not produce progeny or died prior to egg laying were classified as adult sterile. A chi-squared analysis was performed to assess significance.

### Neurodegeneration assays

Strains with *Punc25*::GFP-tagged (GABA)-ergic motor neurons were generated by crossing to the reporter strain CZ1200. Strains were staged to day 1 of adulthood and immobilized on a 2% agarose pad with 0.01% sodium azide. Live VD and DD GABAergic neurons were assessed under fluorescent microscopy on DeltaVision Elite (Applied Precision, Issaquah, WA) imaging system using an Olympus 60× oil objective. The number of live neurons, number of dorsal cord gaps, and percentage of neurons with aberrantly branched neuronal commissures were scored. Statistical significance was analyzed by performing a One-way ANOVA with a Tukey’s post-hoc test using GraphPad Prism statistical software.

### Lifespan analysis

*C. elegans* were synchronized to L4 stage from a timed egg lay on NGM plates at 20 °C. Lifespan plates were prepared from 30 mm NGM plates that were seeded with 10X concentrated OP50 and treated with 10 mg/ml 5-fluorodeoxyuridine (FUDR). One hundred animals per strain were assayed at 25 °C. Animals were checked daily for signs of movement by observing locomotion and pharyngeal pumping. Towards the end of life, animals were tapped lightly with a platinum pick to look for a response. Animals that failed to respond were counted as dead and removed from the plate. Animals that died of bursting, bagging, or mishandling were censored from the data. Statistical significance was analyzed by performing a Chi-squared analysis with a Mantel-Cox test using GraphPad Prism software.

### Immunoblotting

Mixed-stage populations of *C. elegans* were grown on 150 mm 5XPEP plates, washed with M9 buffer, and frozen with liquid nitrogen. Protein lysates were prepared by sonication of frozen *C. elegans* pellets in lysis buffer (10 mM Tris-HCL pH 7.5, 5 mM EDTA, 10% sucrose) at 70% amplitude for 10 s, repeated three times. The lysate was loaded and resolved on precast 4-15% gradient SDS-PAGE gels (BioRad) and transferred to PVDF membrane (Bio-Rad Immun-blot PVDF membrane) at 100 V for 32 m. Human TDP-43 was detected with the commercially available monoclonal antibody ab57105 (Abcam, 1:2500) directed at human TDP-43 amino acids 1-261. TDP-43 phosphorylated at S409/S410 was detected by a commercially available monoclonal antibody (Cosmobio, Catalog # TIP-PTD-M01, 1:1000). Total Tau was detected with a pan-tau polyclonal antibody rb17025 (V. Lee lab, 1:3000) [28]. Tau phosphorylated at T181 was detected by AT270 (Thermo Scientific; 1:15,000). Tau phosphorylated at S202 was detected by CP13 (1:500), a generous gift from Dr. Peter Davies (Albert Einstein College of Medicine, Bronx, NY). Tau phosphorylated at T231 was detected by AT180 (Thermo Scientific, 1:2000). Tau phosphorylated at S396/404 was detected by PHF-1 (P. Davies lab, 1:2000) [29]. Load controls were detected by probing for β-Tubulin as previously described [14].

Human post-mortem brain lysate was prepared by homogenization of tissue in lysis buffer (50 mM HEPES pH 7.5, 1 mM EDTA, 150 mM NaCl, 10% Glycerol, 0.1% Triton X-100, 1 mM PMSF, 1 protease inhibitor pellet (Roche cOmplete Mini)) followed with a 10 s sonication at 50% amplitude using a Branson Sonifier with micro tip. Total protein lysate was loaded in 5XSDS buffer (5% SDS, 200 mM DTT, 50 mM Tris pH 6.8, 5 mM EDTA, 50% sucrose, 0.05% Bromophenol Blue) and resolved on a precast 4-15% gradient SDS-PAGE gel (BioRad) at 200 V and transferred to PVDF membrane (Bio-Rad Immun-blot PVDF membrane) at 100 V for 30 m. TTBK1 was detected with the commercially available polyclonal antibody directed at N-terminal amino acids 240-270 of human TTBK1 (Abcam, ab103944, 1:1000). TTBK2 was detected with the commercially available antibody directed at the N-terminus (Abcam, ab67839).

### Post-mortem human tissue

We obtained de-identified samples of *postmortem* tissue from the University of Washington Alzheimer’s Disease Research Center (ADRC) Neuropathology Core (PI, Dr. C. Dirk Keene) after receiving human subjects approval (University of Washington human subjects division approval: HSD# 06-0492-E/A 01). FTLD cases were selected on the basis of having an autopsy-confirmed diagnosis of FTLD-tau or FTLD-TDP. Control samples were from neurologically healthy control participants, who were of a similar age and were confirmed to be negative for neuropathologic changes of FTLD-tau or FTLD-TDP using routine and immunohistochemical assays. Frontal cortex (prefrontal middle frontal gyrus) and hippocampus (at the level of the lateral geniculate nucleus) samples were dissected at the time of autopsy in coronally sliced brains fixed approximately 3 weeks in 10% neutral buffered formalin according to routine protocols. Samples were processed and embedded in paraffin according to standard protocols.

### Immunohistochemistry and Immunofluorescence

Formalin-fixed, paraffin-embedded human brain tissue samples were sectioned by the University of Washington Alzheimer’s Disease Research Center neuropathology core (Seattle, WA) onto standard charged glass microscope slides. Primary antibodies used for immunohistochemistry were anti-TTBK1 (Abcam, 1:100) and anti-TTBK2 (Abgent, 1:200). In order to minimize variability, sections from all cases (normal and affected subjects) were stained simultaneously for each antibody. Briefly, 5 μm sections from the frontal cortex and hippocampus were deparaffinized in xylene, rehydrated through graded alcohols, and an antigen retrieval step consisting of autoclaving sections in citrate buffer (1.8 mM citric acid/ 8.2 mM sodium citrate) was performed. Sections were treated for endogenous peroxidases with 3% hydrogen peroxide, blocked in 5% milk, incubated with primary antibody overnight at 4 °C, followed by biotinylated secondary antibody for 45 min at room temperature. Finally, sections were incubated in an avidin-biotin complex (Vector’s Vectastain Elite ABC kit, Burlingame, CA) and the reaction product was visualized with 0.05% diaminobenzidine (DAB)/0.01% hydrogen peroxide in PBS. Specificity of these antibodies has been previously shown [9]. Immunohistochemistry photomicrographs were taken with a digital camera and imported into Adobe Photoshop for mounting. To optimize visualization of staining, photomicrographs were modified when necessary by adjusting brightness and contrast.

For double label immunofluorescence experiments, sections were co-immunostained with TTBK1 or TTBK2 and pathological tau as detected by pT231 specific monoclonal antibody AT180 (ThermoScientific). AlexaFluor 647 goat anti-mouse and 568 goat anti-rabbit secondary antibodies (Molecular Probes) were used and autofluorescence was quenched with 0.1% Sudan Black. Microscopy was performed on a Delta Vision microscope (GE, Inc) using a 60× or 100× oil immersion objective, a sCMOS camera, and 2 × 2 binning. Image analysis was performed using softWoRx 6.0 Beta software (GE, Inc). Human brain samples stained for AT180 and TTBK1 or TTBK2 were imaged on a Leica TCS SP5 II confocal microscope using a 63× oil immersion objective. Colocalization analysis of confocal images were conducted in ImageJ 1.51n using Coloc 2.

## Results

### Human TTBK1 and TTBK2 kinase domain transgenic *C. elegans* are behaviorally normal

To understand whether TTBK1 and TTBK2 kinases play a direct role in neurodegeneration, we constructed transgenic (Tg) *C. elegans* lines expressing kinase catalytic domains of human TTBK1 (hTTBK1-cat) or TTBK2 (hTTBK2-cat) under the pan-neuronal promoter *rgef-1* by micro-injecting a plasmid transgene (P*rgef-1*::hTTBK1 and P*regef-1*::hTTBK2). The resultant extrachromosomal transgenic strains were exposed to gamma radiation to create stable genomically integrated transgenic lines. Two lines of each hTTBK1-cat and hTTBK2-cat were then characterized for behavior using a radial locomotion assay. We observed that both hTTBK1-cat lines exhibited hyperactive locomotion when compared with wild type animals (Additional file 1: Figure S1a). We hypothesize the motor hyperactivity is a result of excess kinase activity. Neither TTBK2-cat strain was significantly different from non-transgenic *C. elegans*. One of each hTTBK1-cat and hTTBK2-cat line was selected for further characterization and used for the remainder of experiments. We measured the lifespan of hTTBK1-cat, but saw no significant difference in its median or maximum lifespan as compared to wild-type *C. elegans* (Additional file 1: Figure S1b). Similarly, TTBK2-cat had no significant changes in median or maximum lifespan as compared to wild-type controls (Additional file 1: Figure S1c).

### Co-expression of TTBK1 or TTBK2 with tau causes behavioral abnormalities, aberrant phosphorylation, and shortened lifespan

**Fig. 1:**
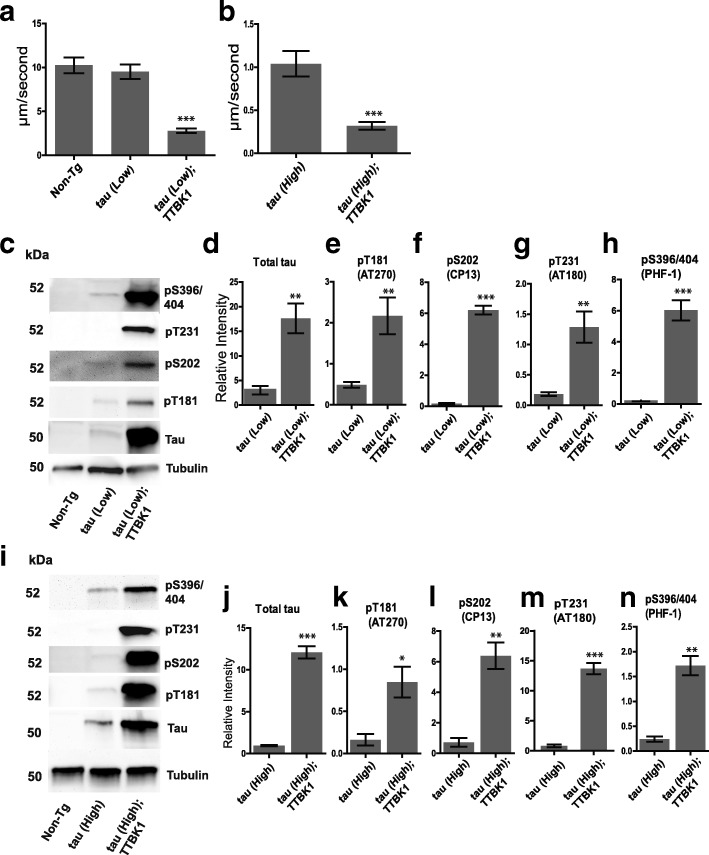
Expression of hTTBK1-cat causes neurodegenerative phenotypes in a tau background. **a** Staged hTTBK1-cat;tau transgenic L4 larvae exhibit significantly decreased radial velocity relative to tau(low) and (**b**) tau(high) transgenic animals. Animals were measured for the linear distance traveled from a central reference point over 1 h, *N* > 70 for each genotype. Significance was determined using an unpaired T-test. *P* < 0.0001 versus tau(low) and *P* < 0.0001 versus tau(high). **c** hTTBK1-cat;tau(low) transgenic animals have increased total tau and ptau relative to tau(low) animals. Bar graphs represent four independent replicate immunoblots of (**d**) Total tau, (**e**) pThr181, (**f**) pSer202, (**g**) pThr231, and (**h**) pSer396/404. Graphs are plotted in relative intensity. Significance was determined using an unpaired T-test. *P* < 0.05 (*), *P* < 0.01 (**), *P* < 0.001 (***). **i** hTTBK1-cat;tau(high) transgenic animals have increased total tau and ptau relative to tau(high) animals. Bar graphs represent four independent replicate immunoblots of (**j**) Total tau, (**k**) pThr181, (**l**) pSer202, (**m**) pThr231, and (**n**) pSer396/404. Graphs represent densitometry analysis and are plotted in relative intensity. Significance was determined using an unpaired T-test. *P* < 0.05 (*), *P* < 0.01 (**), *P* < 0.001 (***)

TTBK1 was originally characterized as a tau kinase and shown to directly phosphorylate tau at Ser198, Ser199, Ser202, and Ser422 in vitro [17]. To test whether TTBK1 driven phosphorylation of tau influences tauopathy phenotypes, we crossed our hTTBK1-cat Tg line with *C. elegans* strains expressing either low or high levels of wild-type human tau (isoform 1N4R). High expression tau Tg worms exhibit a variety of tau-dependent phenotypes including impaired movement, age-dependent neurodegeneration, and accumulation of detergent insoluble phosphorylated tau [14]. The tau(high) Tg line expresses approximately four-fold higher levels of tau than the tau(low) Tg line. Since the tau(high) worms already exhibit a strong phenotype [14], we chose to assess phenotypic changes in a tau(low) line. Tau(low) Tg *C. elegans* do not display significant differences in locomotion as compared to non-transgenic animals, whereas tau(high) Tg animals do exhibit significant impairment. We observed a significant reduction in locomotion velocity as measured by radial dispersion in both hTTBK1-cat;tau(low) (70.7% reduction) and hTTBK1-cat;tau(high) (69.4% reduction) compared to the respective tau transgenes alone (Fig. 1a-b). We also observed a significant increase in both total tau and phosphorylated tau at Thr181, Ser202, Thr231, and Ser396/404 in hTTBK1-cat;tau(low) and hTTBK1-cat;tau(high) strains (Fig. 1c-n). Furthermore, ablation of the active site of the hTTBK1_mut transgene did not modify tau toxicity as measured by motor function (Additional file 1: Figure S1d).

**Fig. 2:**
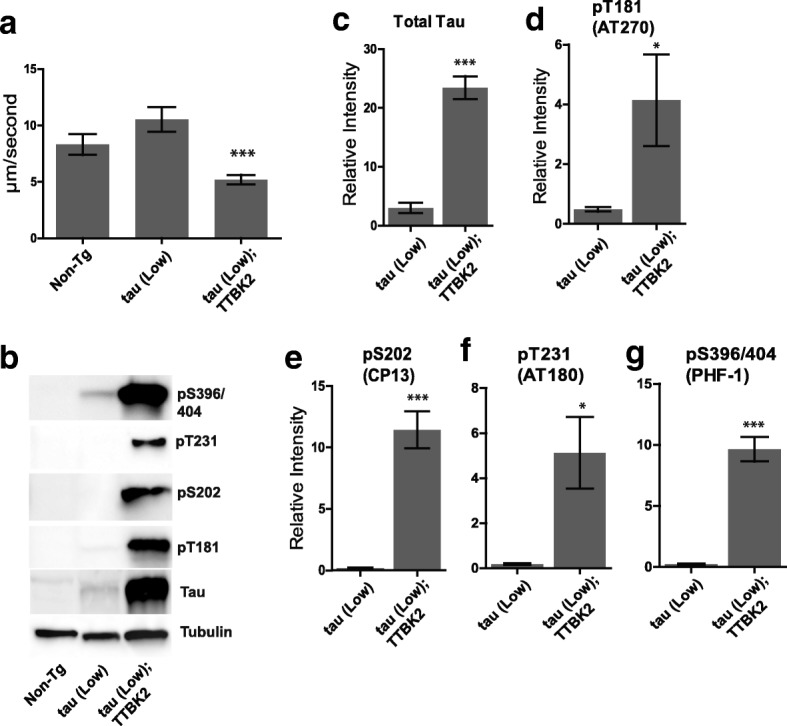
Expression of hTTBK2-cat causes neurodegenerative phenotypes in a tau background. **a** Staged hTTBK2-cat;tau(low) transgenic L4 larvae exhibit significantly decreased radial velocity relative to tau(low) and (**b**) tau(high) transgenic animals. Animals were measured for the linear distance traveled from a central reference point over 1 h, *n* > 75 for each genotype. Significance was determined using an unpaired T-test. *P* < 0.0001. (**b**) hTTBK2-cat;tau transgenic animals have increased total tau and ptau relative to tau animals. Bar graphs represent four independent replicate immunoblots of (**c**) Total tau, (**d**) pThr181, (**e**) pSer202, (**f**) pThr231, and (**g**) pSer396/404. Graphs represent densitometry analysis and are plotted in relative intensity. Significance was determined using an unpaired T-test. *P* < 0.05 (*), *P* < 0.01 (**), *P* < 0.001 (***)

To assess whether TTBK2 catalytic activity could drive tauopathy related phenotypes, we crossed hTTBK2-cat Tg *C. elegans* lines with tau(low) and tau(high) Tg lines. We asked whether TTBK2-cat activity could modulate tau-induced behavioral defects and increase protein phosphorylation. We found that hTTBK2-cat expression exacerbates behavioral defects as indicated by a significant 50.6% reduction in radial motor velocity in our tau(low) Tg lines (Fig. 2a). Additionally, we saw a significant increase in total tau and significant increases phosphorylated tau at Thr181, Ser202, Thr231, and Ser396/404 (Fig. 2b-g).

Given the motor behavior and protein phosphorylation phenotypes in our double transgenic hTTBK1-cat;tau and hTTBK2-cat;tau *C. elegans*, we wanted to test whether there was an effect on longevity. Tau worms lived to a median age of 14 days post-development, whereas hTTBK1-cat;tau worms lived to 12 days post-development and hTTBK2-cat;tau worms lived to 11 days post-development. This equates to a significant 14% reduction in lifespan for hTTBK1-cat;tau and a significant 21% reduction in lifespan for hTTBK2-cat;tau transgenic animals (Additional file 1: Figure S2a). These findings suggest the tauopathy modification is strong enough to limit lifespan as a consequence of synergy between TTBK1 or TTBK2 and tau.

### Co-expression of high levels of tau and hTTBK2 causes lethality

When crossing hTTBK2-cat with tau(high) Tg lines, we were unable to obtain a homozygous hTTBK2-cat;tau(high) population. To determine whether the two transgenes were synthetic lethal in combination, we counted the observed phenotypes of progeny in comparison to expected Mendelian ratios. We generated lines homozygous for tau(high) and heterozygous for hTTBK2-cat (tau(high)/tau(high); hTTBK2-cat/+). These animals were allowed to self-fertilize and the resultant F2 progeny were scored for the presence of a fluorescent co-injection marker to infer the genotype of the F1 parent (hTTBK2-cat/hTTBK2-cat, hTTBK2-cat/+, or +/+). Only 3.6% (6/165) of progeny were homozygous for both tau and TTBK2-cat, compared to the expected Mendelian outcome of 25% for segregation of a single genetic trait in the F1 generation (Table 1). Of those individual populations that were homozygous, 100% of subsequent progeny were dead as larvae. Additionally, we observed that 28.5% (47/165) of F1s were dead as larvae and 19.4% (32/165) of F1s that reached adulthood died prior to egg laying or were sterile. To confirm the results, we repeated the experiment with a second independent hTTBK2 line, and made the parental genotype homozygous for hTTBK2-cat and heterozygous for tau(high) (hTTBK2-cat/hTTBK2-cat; tau(high)/+). Similar to our previous results, only 7.1% (18/254) of F1 individuals were homozygous for both hTTBK2-cat and tau(high) expression with 100% of subsequent F2 progeny observed dead as larvae. Additionally, 18.1% (46/254) of F1s were dead as larvae and of those that developed to adulthood, 11.8% (30/254) were sterile (Table 1). Based on these results, we conclude that TTBK2 kinase activity causes synthetic lethality when co-expressed with high levels of human tau.

**Table 1:** Expression of TTBK2-cat is embryonic lethal in a high-expression tau homozygous background

(Homozygous for TTBK2)	Observed	Expected
tau +/+	6 (3.6%)	41 (25%)
tau +/−	30 (18.2%)	83 (50%)
tau −/−	50 (30.3%)	41 (25%)
Larval Dead	47 (28.5%)	0 (0%)
Adult Sterile	32 (19.4%)	0 (0%)
Total	165	
(Homozygous for tau)	Observed	Expected
TTBK2 +/+	18 (7.1%)	63.5 (25%)
TTBK2 +/−	99 (39.0%)	127 (50%)
TTBK2 −/−	61 (26.5%)	63.5 (25%)
Larval Dead	46 (18.1%)	0 (0%)
Adult Sterile	30 (11.8%)	0 (0%)
Total	254	

### Co-expression of TTBK1 or TTBK2 with tau causes neurodegeneration

To determine whether hTTBK1-cat;tau(high) and hTTBK2-cat;tau(low) lines exhibit normal embryonic neuronal development of GABAergic motor neurons, we crossed our hTTBK1-cat;tau(high) and hTTBK2-cat;tau(low) transgenic animals to a strain carrying a fluorescent GABAergic neuronal reporter driven by the glutamic acid decarboxylase (GAD) promoter (P*unc25*::GFP) [27]*.* Using this reporter, we can assess neuron loss and axonal integrity in living animals with fluorescent microscopy. We scored living GFP-tagged neurons in hTTBK1-cat;tau(high) and hTTBK2-cat;tau(low) developing larvae. We found that all transgenic *C. elegans* appear grossly developmentally normal and all expected GABAergic neurons were present and structurally sound at larval stage L1 (Additional file 1: Figure S3a-f). Additionally, we observed that expression of hTTBK1-cat or hTTBK2-cat does not affect GFP expression levels (Additional file 1: Figure S3g).

**Fig. 3:**
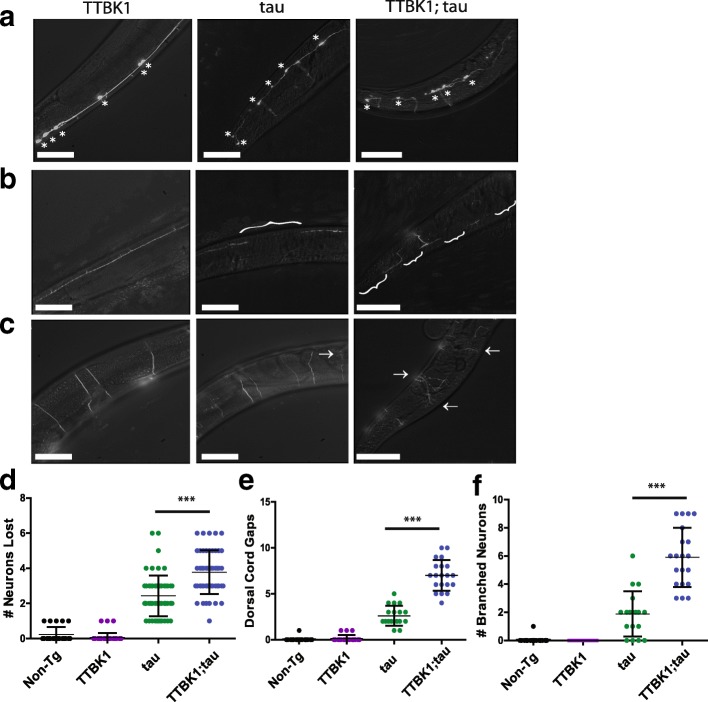
Expression of hTTBK1-cat causes tau-dependent neuron loss and axonal abnormalities. GFP-labeled D-type GABAergic motor neurons were observed in vivo in live day 1 adult transgenic *C. elegans*. **a** Fluorescent images of GABAergic live neurons in the posterior region. Each live neuron is marked with an asterisk. **b** Fluorescent images of GABAergic dorsal cord. Gaps in dorsal cord are marked with brackets (**c**) Fluorescent images of GABAergic axonal commissures. Aberrantly branched commissures are marked with arrows. **d** Number of neurons lost for each worm is plotted. GFP-labeled controls lost an average of 0.22 neurons per animal (*n* = 22). hTTBK1-cat Tg animals lost an average of 0.06 neurons (*n* = 46). Tau Tg animals lost an average of 2.4 neurons (*n* = 56). hTTBK1-cat;tau Tg animals lost an average of 3.8 Neurons (*n* = 49). *P* < 0.001 for tau versus hTTBK1-cat;tau. **e** Number of dorsal cord gaps for each worm is plotted. hTTBK1-cat Tg animals had an average of 0.14 gaps (*n* = 21). Tau Tg animals had an average of 2.6 gaps (*n* = 18). hTTBK1-cat;tau Tg animals had an average of 7.4 gaps (*n* = 10). *P* < 0.001 for tau versus hTTBK1-cat;tau (**f**) Number of aberrantly branched commissures for each worm is plotted. hTTBK1-cat Tg animals had an average of 0 aberrantly branched neurons (*n* = 20). Tau Tg animals had an average of 1.9 aberrantly branched neurons (*n* = 18). hTTBK1-cat;tau Tg animals had an average of 5.9 aberrantly branched neurons (*n* = 20). *P* < 0.001 for tau versus TTBK1-cat;tau. Significance was determined using a one-way analysis of variance with Tukey’s multiple comparison test among strains. Scale bar = 50 μm

Previous evidence suggests phosphorylated tau is increased in neurodegenerative diseases and correlates with the formation of toxic tau aggregates [12, 13, 15]. We tested whether TTBK1/2 driven increases in tau phosphorylation cause neuron loss using the *C. elegans* strains described above. In our hTTBK1-cat;tau(high) animals, we observed a significant decrease in live GABAergic neurons in day 1 adult worms. Live hTTBK1-cat;tau(high) animals lost on average 20% (3.8/19) of neurons as compared to 12.6% (2.4/19) of neurons lost in tau animals by day one of adulthood (Fig. 3a and d). We also assessed whether neuronal connectivity degenerates in these animals by counting gaps in the continuity of the dorsal nerve cord. Whereas a non-transgenic animal presents no degeneration of the nerve cord in early adulthood, we found that hTTBK1-cat;tau(high) animals had significant disruption of nerve cord continuity with on average 7.0 dorsal cord gaps, as compared to tau(high) alone with an average of 2.6 gaps (Fig. 3b and e). Lastly, in our double transgenic hTTBK1-cat;tau(high) worms, there was strong evidence for aberrant axonal branching. In non-transgenic animals, healthy GABAergic motor neurons do not form branches. To quantify this observation, we counted the number of live neurons that exhibited abnormal branching. Our hTTBK1-cat;tau(high) line had on average 31% of GABAergic motor neurons with aberrant branching per worm as opposed to the tau worms, which only had 10% of the same neurons displaying aberrant branching (Fig. 3c and f).

**Fig. 4:**
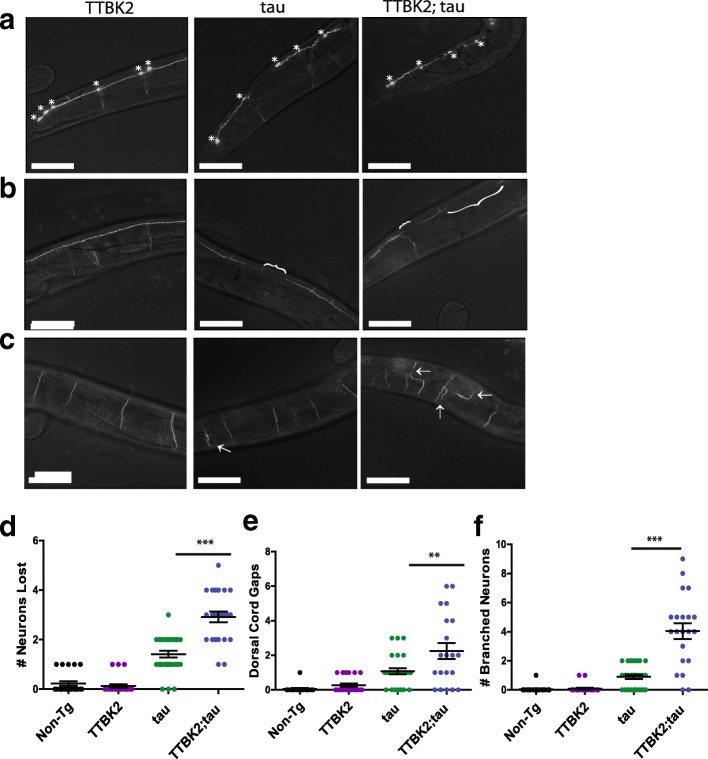
Expression of hTTBK2-cat causes tau-dependent neuron loss and axonal abnormalities. GFP-labeled D-type GABAergic motor neurons were observed in day 1 adult transgenic animals in vivo. **a** Fluorescent images of GABAergic live neurons in the posterior region. Each live neuron is marked with an asterisk. **b** Fluorescent images of GABAergic dorsal cord. Gaps in dorsal cord are marked with brackets (**c**) Fluorescent images of GABAergic axonal commissures. Aberrantly branched commissures are marked with arrows. **d** Number of neurons lost for each worm is plotted. hTTBK2-cat Tg animals lost an average of 0.13 neurons (*n* = 23). Tau Tg animals lost an average of 1.4 neurons (*n* = 29). hTTBK2-cat;tau Tg animals lost an average of 2.9 Neurons (*n* = 23). *P* < 0.001 for tau versus hTTBK2-cat;tau (**e**) Number of dorsal cord gaps for each worm is plotted. hTTBK2-cat Tg animals had an average of 0.27 gaps (*n* = 22). Tau Tg animals had an average of 1.1 gaps (*n* = 28). hTTBK2-cat;tau Tg animals had an average of 2.3 gaps (*n* = 20). *P* < 0.001 for tau versus hTTBK2-cat;tau (**f**) Number of aberrantly branched commissures for each worm is plotted. hTTBK2-cat Tg animals had an average of 0.09 aberrantly branched neurons (*n* = 23). Tau Tg animals had an average of 0.9 aberrantly branched neurons (*n* = 29). hTTBK2-cat;tau Tg animals had an average of 4.1 aberrantly branched neurons (*n* = 21). *P* < 0.001 for tau versus hTTBK2-cat;tau. Significance was determined using a one-way analysis of variance with Tukey’s multiple comparison test among strains. Scale bar = 50 μm

To investigate the role of TTBK2 in tau-mediated neurodegeneration, we also assessed whether hTTBK2-cat;tau(low) animals exhibited increased neuronal loss, dorsal cord degeneration, and altered axonal architecture. We found that in our hTTBK2-cat;tau(low) models there was also a significant loss of GABAergic motor neurons. The hTTBK2-cat;tau(low) animals had lost on average 15% of their GABAergic neurons by day one of adulthood while animals that expressed tau alone lost on average 7% neurons. Furthermore, we observed a significant increase in dorsal cord degeneration of our double transgenic line (2.3 gaps) in comparison to those expressing tau alone (1.1 gaps) as well as significantly more aberrant axonal branching in our hTTBK2-cat;tau(low) lines (21%, 4 branched neurons) versus the tau line (5%, 0.89 branched neurons) (Fig. 4a-f). Together, these data suggest increased neurodegeneration and neuronal dysfunction driven by TTBK1 and TTBK2 in tau transgenic *C. elegans*.

### Co-expression of TTBK1, but not TTBK2 kinase domains with TDP-43 causes behavioral abnormalities and increased phosphorylated TDP-43

**Fig. 5:**
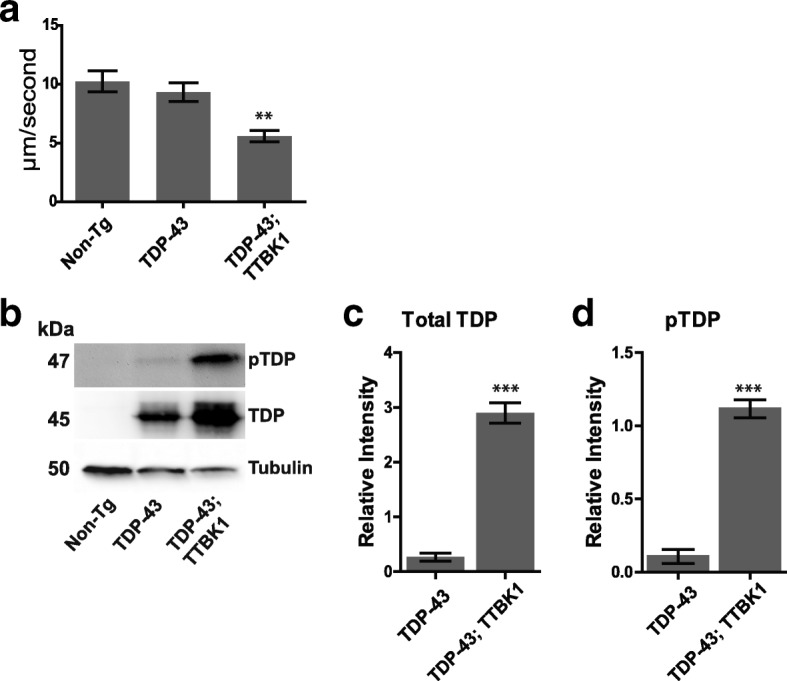
Expression of hTTBK1-cat causes neurodegenerative phenotypes in a TDP-43 background. **a** Staged hTTBK1-cat; TDP-43 transgenic L4 larvae exhibit significantly decreased radial velocity relative to TDP-43 transgenic animals. Animals were measured for the linear distance traveled from a central reference point over 1 h, *N* > 90 for each genotype. Significance was determined using an unpaired T-test. *P* < 0.01 versus TDP-43. **b** hTTBK1-cat; TDP-43 transgenic animals have increased total TDP-43 and pTDP-43 relative to TDP-43 animals. Bar graphs represent six independent replicate immunoblots of (**c**) Total TDP and (**d**) pTDP. Graphs are plotted in relative intensity. Significance was determined using an unpaired T-test. *P* < 0.05 (*), *P* < 0.01 (**), *P* < 0.001 (***)

Given that human TTBK1 phosphorylates TDP-43 at S409/410 in vitro and colocalizes with pathological TDP-43 in both ALS and FTLD post-mortem tissues [9], we wanted to observe the effects of TTBK1 activity on TDP-43 in vivo. We crossed our hTTBK1-cat transgenic *C. elegans* to hTDP-43 transgenic models expressing wild-type TDP-43 [8] to generate hTTBK1-cat;TDP-43 transgenic animals. Animals expressing wild-type TDP-43 alone do not exhibit motor defects as compared to non-transgenic animals. We observed a significant decrease in hTTBK1;TDP-43 locomotion as measured by radial dispersion velocity relative to TDP-43 alone (Fig. 5a). We then asked whether TTBK1 activity influenced accumulation or phosphorylation of TDP-43. We found a significant increase in both total TDP-43 and S409/410 phosphorylated TDP-43 (Fig. 5b-d) in our double transgenic lines. Given the apparent effects on pathological TDP-43 protein accumulation and the impairment of motor phenotypes in our double transgenic hTTBK1-cat;TDP-43 *C. elegans*, we wanted to test whether there was also an effect on longevity. However, we found no significant difference in lifespan when compared to the TDP-43 alone (Additional file 1: Figure S2b). Likewise, hTTBK1_mut transgenes with a disrupted active site failed to modify TDP-43 toxicity (Additional file 1: Figure S1d).

We also asked whether expression of hTTBK2-cat in a TDP-43 background produces similar phenotypes by crossing our hTTBK2-cat *C. elegans* line to wild-type hTDP-43 transgenic *C. elegans*. Although there was a slight increase in total TDP-43 and phosphorylated TDP-43 levels, we did not observe a significant change in behavior or phosphorylated TDP-43 accumulation in the hTTBK2-cat;TDP-43 double transgenic (Additional file 1: Figure S4), demonstrating that hTTBK2-cat likely does not influence TDP-43 toxicity in vivo as strongly as hTTBK1-cat.

### TTBK1 levels are elevated in both FTLD-tau and FTLD-TDP

**Fig. 6:**
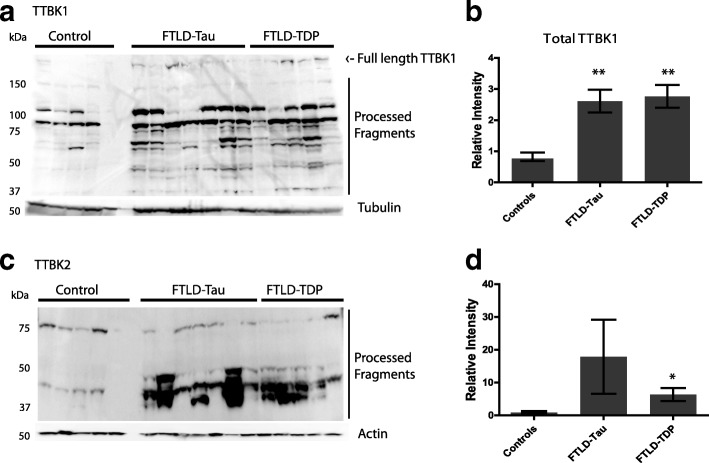
FTLD cases exhibit increased levels of TTBK fragments. **a** Both FTLD-tau and FTLD-TDP patients exhibit significantly increased full-length TTBK1 and processed TTBK1 expression as compared to age-matched controls. **b** Bar graphs represent quantification of all TTBK1 bands present plotted in relative intensity. Significance was determined using a one-way ANOVA. *P* < 0.001 (**). **c** Both FTLD-tau and FTLD-TDP patients exhibit significantly increased processed TTBK2 kinase domain expression as compared to age-matched controls. **d** Bar graphs represent quantification of all TTBK2 bands present plotted in relative intensity. Significance was determined using a one-way ANOVA

We have demonstrated in *C. elegans* that catalytically active TTBK1 and TTBK2 promote tau and TDP-43 phosphorylation [9]. However, it is unknown whether there are changes in the abundance of TTBK1 and TTBK2 in patients with FTLD. In order to assess whether elevation of TTBK1 or TTBK2 occurs in FTLD, we examined TTBK1 and TTBK2 protein levels in human post-mortem brain tissues of both FTLD-tau and FTLD-TDP patients. To measure changes in protein abundance, we performed immunoblot analyses on human postmortem cortical tissue. We found that in both FTLD-tau (*n* = 7) and FTLD-TDP (*n* = 5) cases there was a visible increase in the levels of proteolytically processed TTBK1 and TTBK2 kinase domain bearing species as well as full length TTBK1 (Fig. 6a-d).

**Fig. 7:**
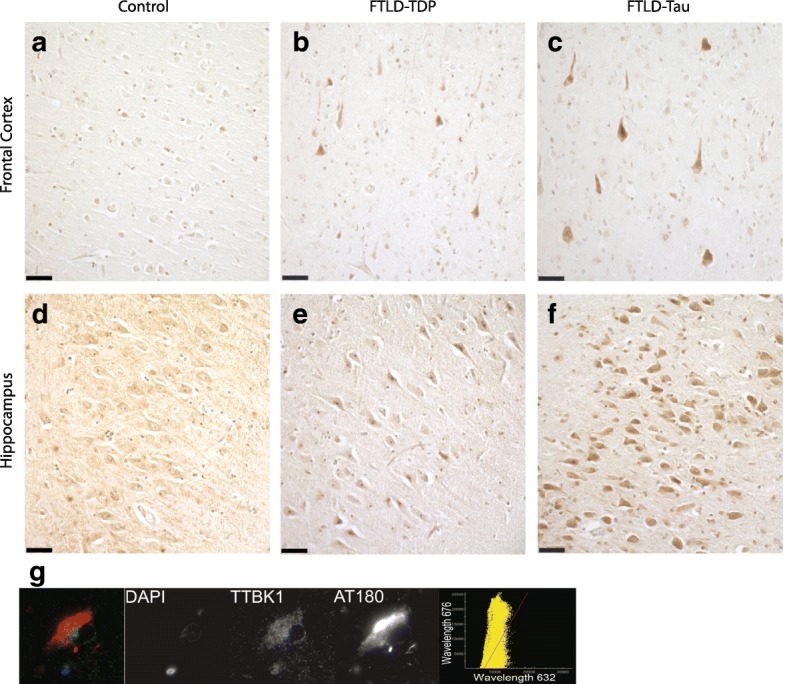
FTLD cases exhibit increased TTBK1 immunostaining. Representative images demonstrating upregulation of TTBK1 immunoreactivity in FTLD brain tissue (*n* = 5 cases of FTLD-TDP represented in panels b,e; *n* = 7 cases of FTLD-tau represented in panels (**c**, **f**)) compared to that of normal control subjects (**a**, **d**). In the hippocampus (**d**-**f**), the increase in TTBK1 is highly robust in FTLD-tau cases relative to both FTLD-TDP cases and controls. In the frontal cortex (a-c) there is enhanced TTBK1 immunoreactivity relative to normal controls, but the difference between the FTLD subtypes was less apparent. Scale bar = 50 μm. **g** TTBK1 is expressed throughout the cytoplasm, and overlaps with phosphorylated tau (AT180) in human cells. Shown is an image from a representative case (*n* = 3). Pearson coefficient of correlation for colocalization = 0.69 ± 0.18 for *n* = 46 neurons analyzed

**Fig. 8:**
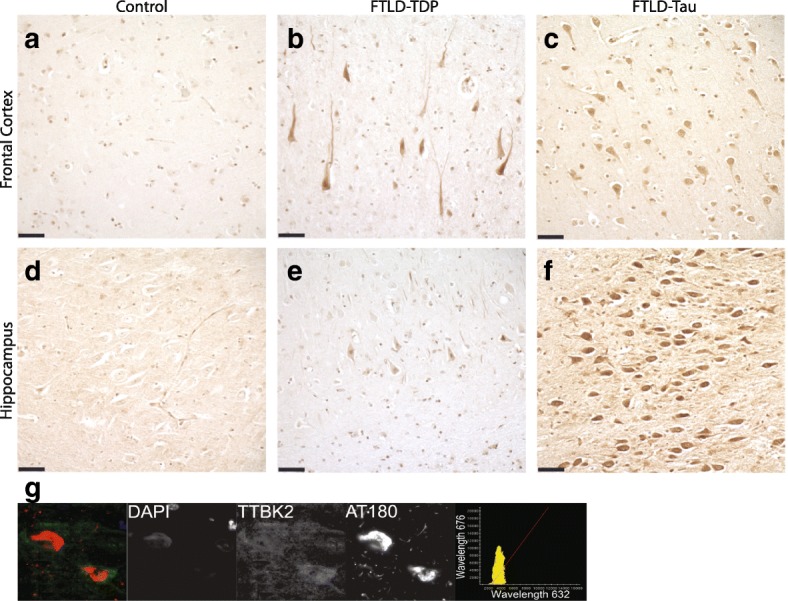
FTLD cases exhibit increased TTBK2 immunostaining. Representative images demonstrating upregulation of TTBK2 immunoreactivity in FTLD brain tissue (*n* = 5 cases of FTLD-TDP represented in panels b,e; 7 cases of FTLD-tau represented in panels (**c**, **f**) compared to that of normal control subjects (**a**, **d**). In the hippocampus (**d**-**f**), the increase in TTBK2 is highly robust in FTLD-tau cases relative to both FTLD-TDP cases and controls. In the frontal cortex (**a**-**c**) there is enhanced TTBK2 immunoreactivity relative to normal controls, but the difference between the FTLD subtypes was less apparent. Scale bar = 50 μm. **g** TTBK2 is expressed throughout the cytoplasm, and overlaps with phosphorylated tau in human cells. Shown is a representative case (*n* = 3). Pearson coefficient of correlation for colocalization = 0.57 ± 0.18 for *n* = 32 neurons analyzed

To establish the distribution of TTBK1/2 accumulation, we examined TTBK1 and TTBK2 immunoreactivity in the hippocampus and frontal cortex of FTLD-tau and FTLD-TDP cases by immunohistochemistry. Our previous study demonstrated an increase in TTBK1 and TTBK2 immunostaining in the frontal cortex of FTLD-TDP cases [9]. In this study, we examined additional FTLD-TDP cases and extended our analyses to include the hippocampus. We again observed an increase in TTBK1 and TTBK2 immunostaining in the frontal cortex of FTLD-TDP cases relative to normal controls (Figs. 7b and 8b). In contrast, immunoreactivity in the hippocampus of FTLD-TDP cases was similar to normal control cases (Figs. 7e and 8e). In FTLD-tau cases, we observed increased TTBK1 and TTBK2 immunoreactivity in both the frontal cortex and hippocampus relative to normal controls (Fig. 7c and f; Fig. 8c and f). The increase in TTBK1/2 immunostaining in the hippocampus of FTLD-tau cases is strikingly robust, especially in CA3 pyramidal neurons, and is more pronounced relative to the increase observed in the frontal cortex of FTLD-TDP.

We previously showed that TTBK1 and TTBK2 co-localize with phosphorylated TDP-43 in cytoplasmic inclusions in FTLD-TDP cases. To determine whether TTBK1/2 co-localize with phosphorylated tau in FTLD-tau cases, we performed double label immunofluorescence on hippocampal sections (Figs. 7g and 8g). We observed TTBK1/2 immunofluorescence throughout the cytoplasm of hippocampal pyramidal neurons that overlapped with AT180 immunofluorescence in neurons positive for these pathological tau deposits. In general, the co-localization of TTBK1 with this phosphorylated tau species was stronger than that of TTBK2. Taken together with our findings in human brain tissue, this suggests that elevated levels of TTBK1 and TTBK2 proteins are present in patients with both FTLD-tau and FTLD-TDP and co-localize with phosphorylated protein.

## Discussion

Previous work has shown that TTBK1 and TTBK2 phosphorylate both tau [17, 25] and TDP-43 [30]. However, the full extent to which tau and TDP-43 are phosphorylated by TTBK1 and TTBK2 in vivo have yet to be described. It remains unknown whether changes in TTBK1 and TTBK2 activity, abundance, and proteolytic processing influence tau- and TDP-43-proteinopathies. To address these gaps in knowledge, we generated transgenic *C. elegans* expressing active human TTBK1 and TTBK2 kinase domains, and evaluated their effects on tau and TDP-43 transgenic models of FTLD. We also characterized TTBK1 and TTBK2 expression patterns and levels in both FTLD-tau and FTLD-TDP subtypes.

We found that hTTBK1-cat expression dramatically increased total and phosphorylated protein levels of human tau and TDP-43 in transgenic *C. elegans* resulting in exacerbated behavioral phenotypes and neurodegeneration. Interestingly, while hTTBK2-cat expression drove accumulation of total and phosphorylated tau and behavioral defects in tau transgenic animals, hTTBK2-cat was relatively neutral in TDP-43 transgenic animals. This suggests an in vivo selectivity of TTBK1 and TTBK2 towards their phosphorylation targets that differs from their in vitro ability to phosphorylate purified TDP-43. Furthermore, these data could also reflect regional or neuronal subtype selectivity by TTBK2.

Although increases in TTBK1 expression have been previously shown in human AD cases [16], there have been no studies examining changes in TTBK1 or TTBK2 abundance in FTLD. In this study we show that both TTBK1 and TTBK2 protein levels increase as compared to age-matched controls in FTLD-TDP and FTLD-tau cases. These findings were seen in both immunoblot analyses and immunohistochemistry studies. These results suggest that changes in TTBK1 and TTBK2 abundance or processing may influence their kinase activities towards tau and TDP-43 in FTLD-tau and FTLD-TDP.

Importantly, this study suggests distinct target selectivity between TTBK1 and TTBK2 kinase activity despite high homology between the kinase domains (88% identity and 96% similarity) [24]. The differential regulation of TTBK1 vs TTBK2 activity is poorly understood. However, here we show that TTBK2 kinase domain has a greater influence on promoting tau-induced neurodegeneration than TTBK1, but it does not appear to affect TDP-43 in vivo. Likewise, TTBK1 is able to phosphorylate both TDP-43 and tau in vivo, but appears to have a relatively modest effect on tau compared to TTBK2. Investigations into the regulation of TTBK1 and TTBK2 kinase activity and substrate specificity are important next steps in determining the roles of TTBK1 and TTBK2 in FTLD.

## Conclusions

The identification of TTBK1 and TTBK2 as both tau and TDP-43 kinases indicates a possible shared mechanism for the initiation of TDP-43 proteinopathy and tauopathy in FTLD. This is supported by the pathological presence of either phosphorylated TDP-43 or tau in the majority of FTLD cases, and the elevated protein expression of TTBK1 and TTBK2 in both FTLD-tau and FTLD-TDP. Therefore, the development of drugs to selectively inhibit TTBK1 and TTBK2 may be a common therapeutic strategy for both FTLD-tau and FTLD-TDP. In general, kinases have become among one of the most important classes of drug targets [31]. TTBK1 in particular makes an attractive drug target due to its restricted expression in neurons [24], unlike other tau kinases such as GSK3 and CDK5 that are ubiquitously expressed [32, 33]. Furthermore, TTBK1 is one of the first kinases to be identified as both a tau and TDP-43 kinase and our results suggest that TTBK1 kinase activity is able to induce neurodegenerative phenotypes in both tau and TDP-43 backgrounds. TTBK1 selective kinase inhibitors therefore represent a potential means to treat both FTLD-tau and FTLD-TDP.

## Additional file

Additional file 1: Supplemental Data_11_17_17_.pdf. (PDF 1301 kb)

## Abbreviations

AD: Alzheimer’s disease
ADRC: Alzheimer’s Disease Research Center
ALS: Amyotrophic Lateral Sclerosis
CBD: Corticobasal Degeneration
CNS: Central Nervous System
FTLD: Frontotemporal Lobar degeneration, −tau: with tau pathology, -TDP: with TDP-43 pathology
FUDR: 5-fluorodeoxyuridine
GAD: Glutamic Acid Decarboxylase
hTTBK1_mut: Human TTBK1 catalytic-dead mutant
hTTBK1-cat: Human TTBK1 catalytic domain
hTTBK2-cat: Human TTBK2 catalytic domain
NGM: Nematode Growth Media
PSP: Progressive Supranuclear Palsy
TDP-43: Trans Activating Response DNA-binding protein
Tg: Transgenic
TTBK1/2: Tau-Tubulin Kinases 1 and 2
