# Supplementary material for: Pathological phosphorylation of tau and TDP-43 by TTBK1 and TTBK2 drives neurodegeneration
Source: Mol Neurodegener. 2018 Feb 6;13:7. doi: 10.1186/s13024-018-0237-9 (PMC5802059; doi:10.1186/s13024-018-0237-9)
Supplement: Supplementary file 1 — Supplemental Data_11_17_17_.pdf. (PDF 1301 kb) [file 13024_2018_237_MOESM1_ESM.pdf]

Supplemental Figure 1: Characterization of hTTBK-cat *C. elegans* lines

(a) Synchronized TTBK-cat transgenic L4 larvae behavior was measured by radial velocity relative to non-transgenic (non-Tg) animals. Animals were measured for the linear distance traveled from a central reference point over one hour, N>75 for each genotype. Significance was determined using an unpaired T-test. P=0.04 for Non-TG vs. hTTBK1-cat and P= 0.007 for Non-Tg vs. hTTBK1-cat. Lines that were selected for further analysis are indicated by black diamonds. (b) Lifespan analysis for hTTBK1-cat. (c) Lifespan analysis for hTTBK2-cat. (d) Extrachromasomal array transgenic TTBK1 kinase dead mutants crossed with Tau transgenic animals. Note that TTBK1 kinase dead mutants have normal locomotion relative to non-tg and do not significantly change tau mediated locomotion defects. (e) Kinase Dead TTBK1 transgenes crossed with TDP-43 transgenic lines. Note that TTBK1 kinase dead mutants do not significantly change TDP-43 mediated locomotion defects.

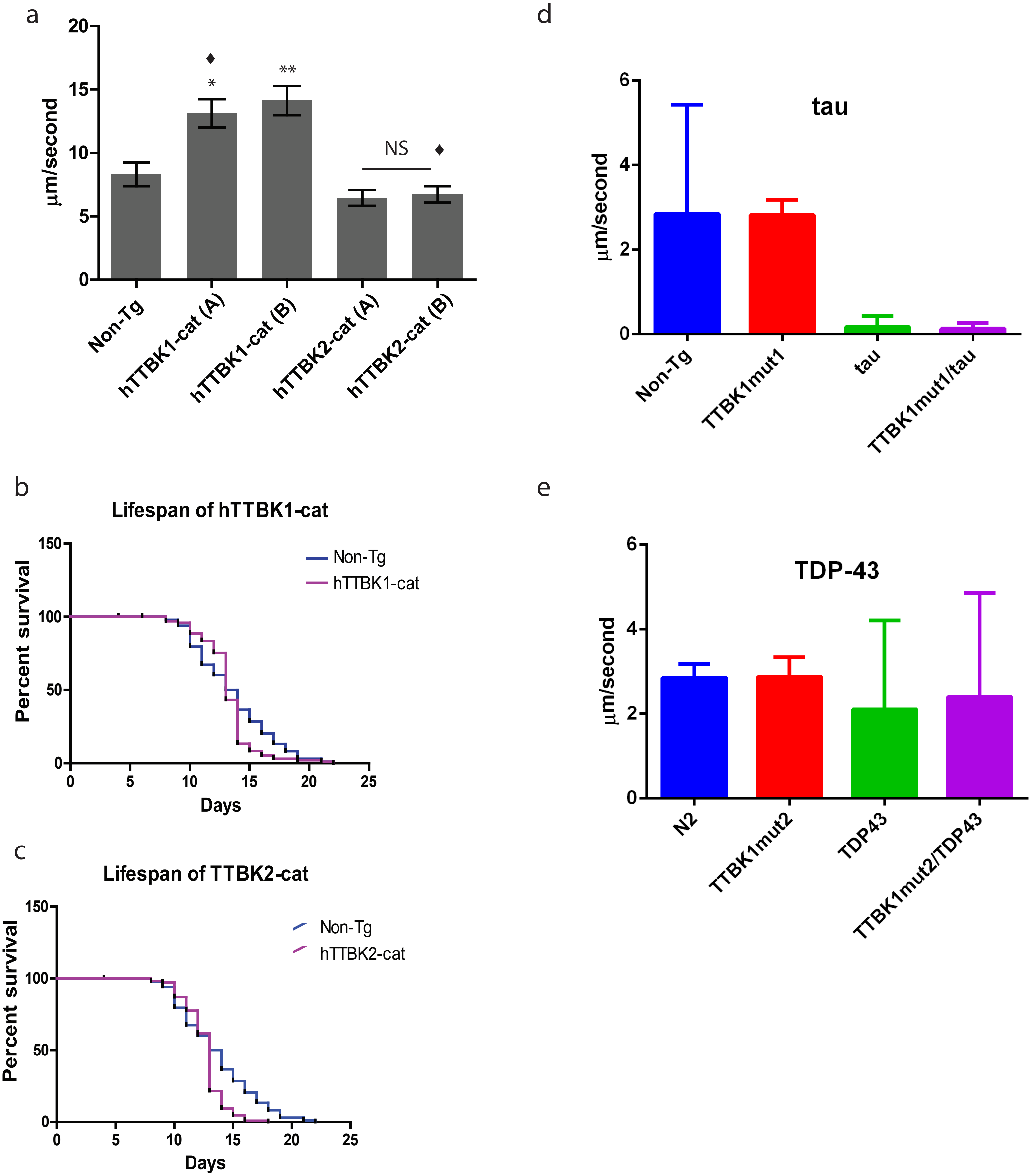

a
µm/second
20
15
10
5
0
◆
*
**
NS
◆
Non-Tg
hTTBK1-cat (A)
hTTBK1-cat (B)
hTTBK2-cat (A)
hTTBK2-cat (B)
b
Lifespan of hTTBK1-cat
Percent survival
150
100
50
0
Non-Tg
hTTBK1-cat
0
5
10
15
20
25
Days
c
Lifespan of TTBK2-cat
Percent survival
150
100
50
0
Non-Tg
hTTBK2-cat
0
5
10
15
20
25
Days
d
tau
µm/second
6
4
2
0
Non-Tg
TTBK1mut1
tau
TTBK1mut1/tau
e
TDP-43
µm/second
6
4
2
0
N2
TTBK1mut2
TDP43
TTBK1mut2/TDP43

Supplemental Figure 2: Lifespan analyses

Lifespans of each transgenic line was assessed at 25°C. (a) Lifespan analysis of tau versus tau;TTBK1 or tau;TTBK2. Tau median lifespan = 14 days, tau; TTBK1 median lifespan = 13 days, tau; TTBK2 median lifespan = 12 days. Significance was analyzed using a Chi-squared analysis with a Mantel-Cox test. $P=0.04$ for tau vs. tau; TTBK1 and $P<0.001$ for tau vs. tau; TTBK2. (b) Lifespan analysis of TDP-43 versus TDP-43; TTBK1. TDP-43 median lifespan = 8 days, TDP-43; TTBK1 median lifespan = 7.5 days. No significance.

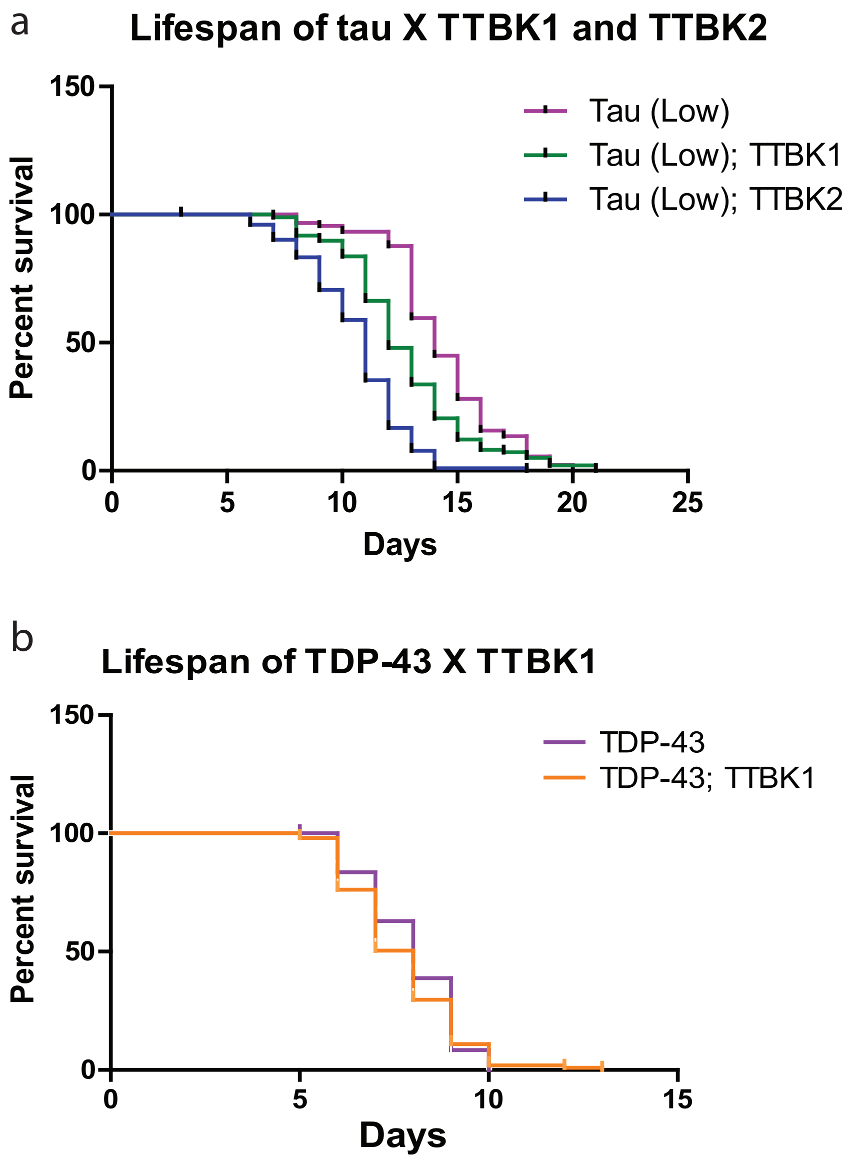
a
Lifespan of tau X TTBK1 and TTBK2
Percent survival
150
100
50
0
0
5
10
15
20
25
Days
Tau (Low)
Tau (Low); TTBK1
Tau (Low); TTBK2
b
Lifespan of TDP-43 X TTBK1
Percent survival
150
100
50
0
0
5
10
15
Days
TDP-43
TDP-43; TTBK1

Supplemental Figure 3: L1 transgenic *C. elegans* are developmentally normal and GFP levels are unaffected by hTTBK1 or hTTBK2 expression

GFP-labeled D-type GABAergic motor neurons were observed in L1 larval transgenic animals *in vivo* living worms. (a) TTBK1 (b) tau (high) (c) tau (high); TTBK1 (d) TTBK2 (e) tau (low) (f) tau (low); TTBK2. Asterisks indicate live neurons. Scale bar=50um. (g) GFP expression levels are not influenced by the presence of hTTBK1-cat or hTTBK2-cat.

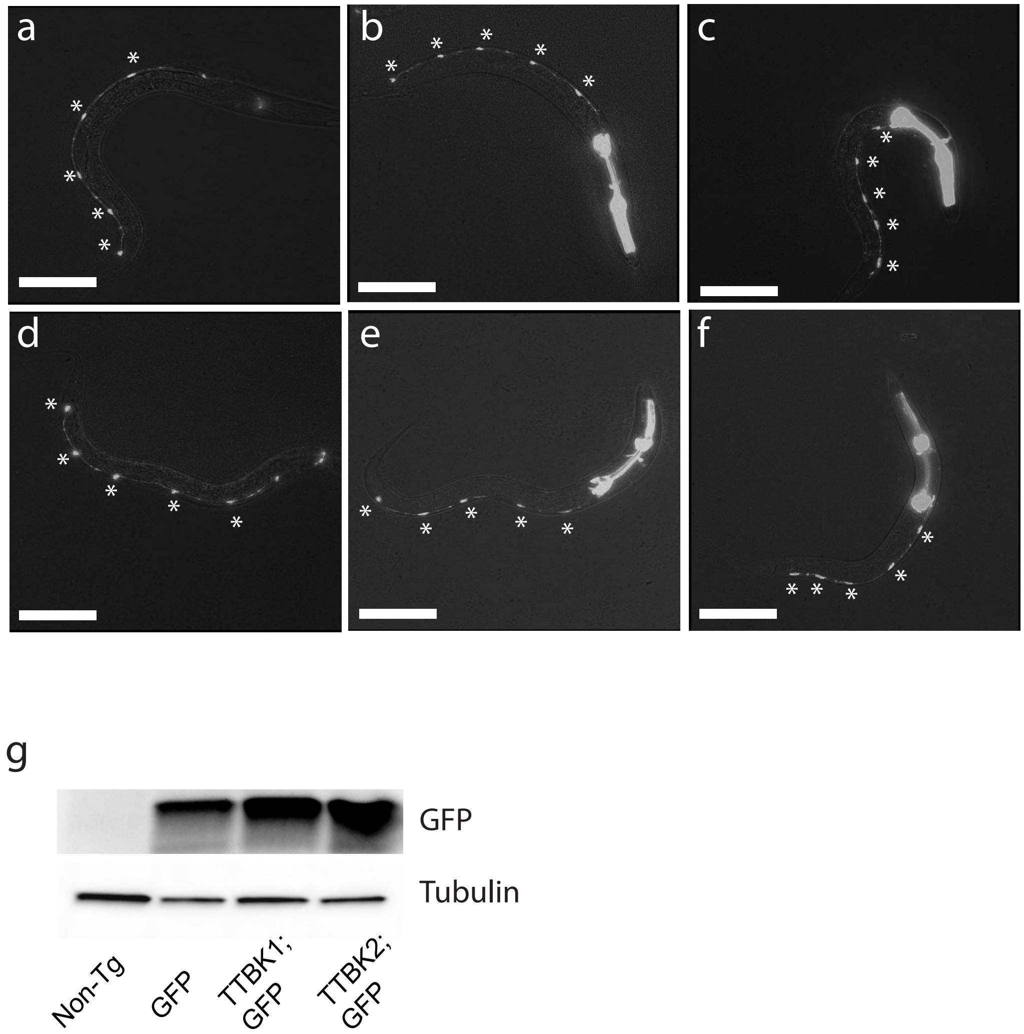

a
b
c
d
e
f
g
GFP
Tubulin
Non-Tg
GFP
TTBK1; GFP
TTBK2; GFP

Supplemental Figure 4: TTBK2 does not affect TDP-43 phenotypes

(a) Staged hTTBK2-cat; TDP-43 transgenic L4 larvae do not exhibit significantly decreased radial velocity relative to TDP-43 transgenic animals. Animals were measured for the linear distance traveled from a central reference point over one hour, N>100 for each genotype. Significance was determined using an unpaired T-test. P=0.13 versus TDP-43. (b) hTTBK2-cat; TDP-43 transgenic animals have slightly increased but not statistically significant total TDP-43 and pTDP-43 relative to TDP-43 animals. Bar graphs represent six independent replicate immunoblots of (c) Total TDP-43 and (d) pTDP. Graphs are plotted in relative intensity. Significance was determined using an unpaired T-test. P=0.059 for total TDP and p=0.13 for pTDP.

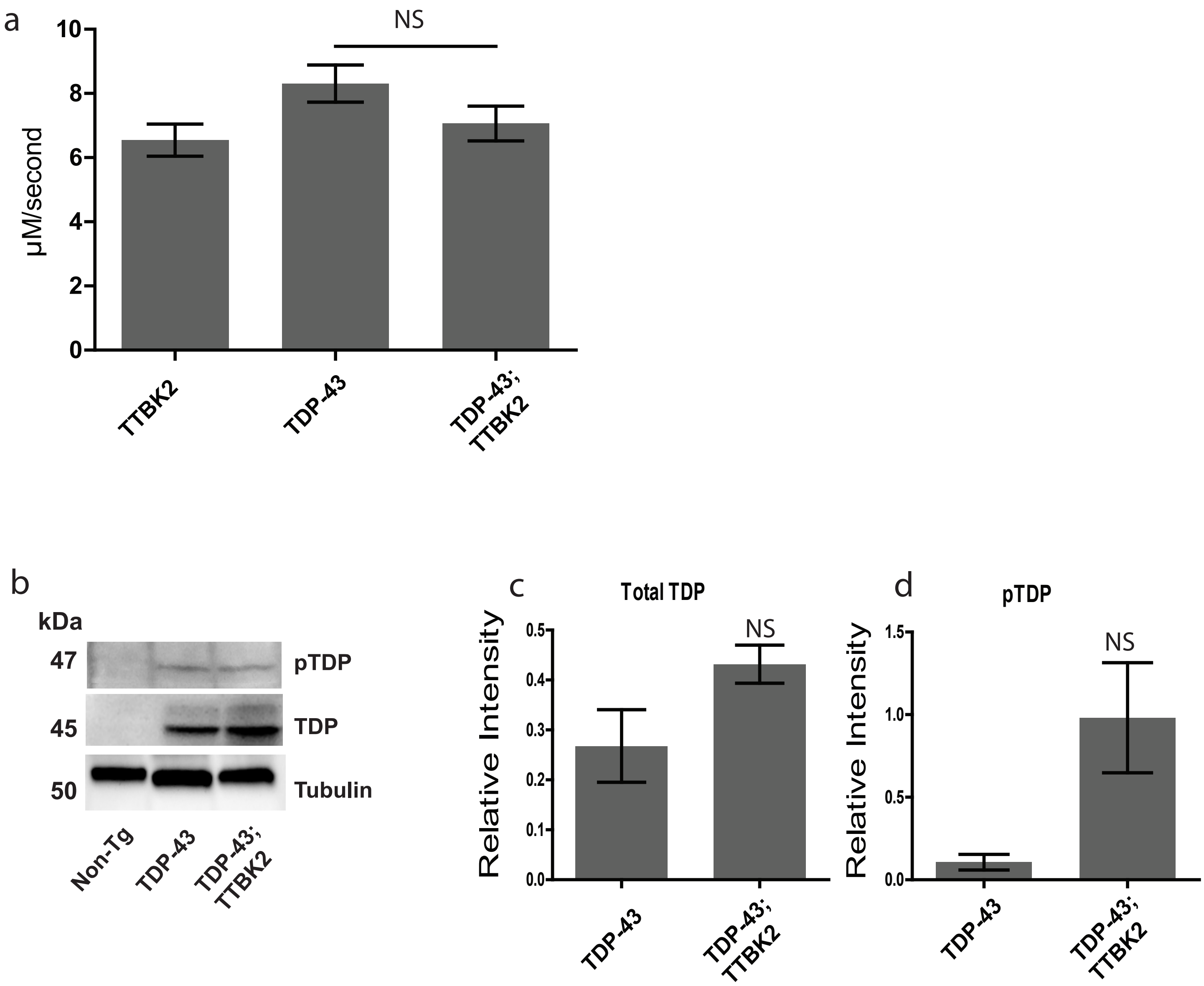
a
NS
μM/second
10
8
6
4
2
0
TTBK2
TDP-43
TDP-43;
TTBK2
b
kDa
47
45
50
pTDP
TDP
Tubulin
Non-Tg
TDP-43
TDP-43;
TTBK2
c
Total TDP
Relative Intensity
0.5
0.4
0.3
0.2
0.1
0.0
NS
TDP-43
TDP-43;
TTBK2
d
pTDP
Relative Intensity
1.5
1.0
0.5
0.0
NS
TDP-43
TDP-43;
TTBK2
